# Evaluation of the extraction of methodological study characteristics with JATSdecoder

**DOI:** 10.1038/s41598-022-27085-y

**Published:** 2023-01-04

**Authors:** Ingmar Böschen

**Affiliations:** Institute of Psychology, Research Methods and Statistics, University Hamburg, Von-Melle-Park 5, 20146 Hamburg, Germany

**Keywords:** Psychology, Environmental social sciences

## Abstract

This paper introduces and evaluates the *study.character* module from the *JATSdecoder* package which extracts several key methodological study characteristics from NISO-JATS coded scientific articles. *study.character* splits the text into sections and applies its heuristic-driven extraction procedures to the text of the method and result section/s. When used individually, *study.character*’s functions can also be applied to any textual input. An externally coded data set of 288 PDF articles serves as an indicator of *study.character*’s capabilities in extracting the number of sub-studies reported per article, the statistical methods applied and software solutions used. Its precision of extraction of the reported $\alpha$-level, power, correction procedures for multiple testing, use of interactions, definition of outlier, and mentions of statistical assumptions are evaluated by a comparison to a manually curated data set of the same collection of articles. Sensitivity, specificity, and accuracy measures are reported for each of the evaluated functions. *study.character* reliably extracts the methodological study characteristics targeted here from psychological research articles. Most extractions have very low false positive rates and high accuracy ($\ge 0.9$). Most non-detections are due to PDF-specific conversion errors and complex text structures, that are not yet manageable. *study.character* can be applied to large text resources in order to examine methodological trends over time, by journal and/or by topic. It also enables a new way of identifying study sets for meta-analyzes and systematic reviews.

## Introduction

In scientific research practice, many individual decisions can be made that affect the scientific quality of a study. There are also changing standards set by journal editors and the community. This applies not only to the study design, but also to the choice of statistical methods and their settings. With new methods and standards, the way research is planned, conducted and presented changes over time and represents an interesting field of research. One aspect to consider is the ever-increasing number of scientific publications coming out each year. Numerous studies have investigated the use and development of statistical techniques in scientific research practice^1–7^. Most of these studies used manually coded data of a limited number of articles, journals, topics or time interval. The selectivity of these samples therefore severely limits the generalizability of the findings to a wider scope. For example, Blanca et al.^7^ analyzed the use of statistical methods and analysis software solutions in 288 articles (36 articles each from 8 journals), all from a publication period of about one year.

A technology that is suitable for analyzing large amounts of text and helps to overcome the problem of small samples in the analysis of scientific research practice is text mining. Text mining is the process of discovering and capturing knowledge or useful patterns from a large amount of unstructured textual data^8^. It is an interdisciplinary field that draws on data mining, machine learning, natural language processing, statistics, and more^8^. It facilitates extraction and unification tasks that cannot be done by hand when the analyzed text corpus becomes large. In addition to rudimentary computer commands on textual input (regular expressions), there are also many software programs and toolkits that provide model-based methods of natural language processing (NLP).

Well-known NLP libraries such as *NLTK*^9^ or *spaCy*^10^ provide users with a variety of programs for linguistic evaluation of natural language. This often involves the use of statistical models and machine learning. In contrast, the *JATSdecoder* package^11^ focuses on metadata and study feature extraction (in the context of the NISO-JATS format). This extraction is implemented using expert-driven heuristics. Thus, unlike in the aforementioned large multipurpose NLP libraries, no further programming effort is required to perform specific extraction.

Research on scientific practice can benefit greatly from NLP techniques. Compared to manual coding, an automated identification of study characteristics is very time and cost-efficient. It enables large-scale and trend analyzes, mirroring of scientific research practices and identification of studies that meet certain methodological requirements for meta-analyses and systematic reviews. In addition, automated plausibility checks and global summaries can support quality management.

In general, most methodological study characteristics (e.g., statistical results, $\alpha$-level, power, etc.) are reported in a fairly standard way. Here, the module *study.character* from the R package *JATSdecoder*^11^ is presented and evaluated as a tool for extracting key methodological features from scientific reports. The evaluation of the built-in extraction functions is performed on a medium-sized collection of articles (N = 287) but highlights the possibilities in mirroring and identifying methodological trends in rather big article collections. Although the use of method-based NLP methods might be appropriate for the study features focused here, all functions run fine-tuned expert-driven extraction heuristics to achieve a robust extraction and traceability of errors. While many NLP libraries can be thought of as a toolbox for a variety of problems, *JATSdecoder* represents a precision tool for a specific problem.

## The JATSdecoder package

Scientific research is mostly published in two ways. In addition to a printable version which is distributed as PDF file, machine-readable versions are accessible in various formats (HTML, XML, JSON). The PubMed Central database^12^ currently stores almost five million open access documents from the biology and health sciences, distributed as XML files and structured using the Journal Archiving Tag System NISO-JATS^13^. The NISO-JATS is an HTML tag standard to store scientific article content without any graphical parameters (website layout, text arrangement, etc.), graphical content is hyper referenced.

*JATSdecoder*^11^ is a software package for the statistical programming language R^15^. Its function *JATSdecoder* converts NISO-JATS encoded XML documents into a list with metadata, user-adjustable sectioned text and reference list^16^. The structured list is very useful for costum search and extraction procedures, as it facilitates these tasks on selectively defined text parts (e.g., section headings, method or results section, reference list).

The algorithms of *JATSdecoder* were iteratively developed based on the PubMed Central article collection (at that time $\approx 3$ million native NISO-JATS XML) and more than 10,000 PDF files from different journals that were converted to XML files with the Content ExtRactor and MINEr^14^(*CERMINE*).

*CERMINE* is a sophisticated PDF conversion tool which extracts metadata, full text and parsed references from scientific literature in PDF format. The output can be returned as plain text or NISO-JATS encoded content. Compared to a pure text extraction, the transfer into the NISO-JATS format with *CERMINE* is a great advantage for post-processing. Article metadata can be accessed directly and the text of multi-column blocks is extracted correctly, which is often not the case with the output of other conversion software. Supervised and unsupervised machine learning algorithms enable *CERMINE* to adapt to the different document layouts and styles of the scientific literature. Large file collections can be converted using batch processing. Thus, with the help of CERMINE, the publications of another large group of publishers can be processed with *JATSdecoder*.

In addition to the extraction of metadata and study features, *JATSdecoder* provides some convenient, purely heuristic-driven functions that can be useful for any text-analytic approach. An overview of these functions and their functionality is given in Table 1. All functions are based on the basic R environment and make intense use of regular expressions. *letter.convert()* unifies hexadecimal and many HTML letters into a Unicode representation and corrects most PDF and *CERMINE* specific conversion errors. For example, more than 20 different hexadecimal characters that encode a space are converted to a standard space, invisible spaces (e.g.: ‘u200b’) are removed. When extracting text from PDF documents, special characters can often not be read correctly, as they can be stored in a wide variety of formats. Badly compiled Greek letters (e.g., ‘*v*2’ not ‘$\chi ^2$’) and operators (e.g., ‘5‘ not ‘$=$’) are corrected, a ‘${<}{=}{>}$’ is inserted for missing operators (e.g., ‘t${<}{=}{>}$1.2, p${<}{=}{>}$0.05’ for ‘t 1.2, p 0.05’). These unifications are important for further processing and facilitate text search tasks and extractions. *text2sentences()* converts floating text into a vector of sentences. Many not purely digit-based representations of numbers (words, fractions, percentages, very small/high numbers denoted by $10^x$ or $e+x$) can be converted to decimals with *text2num()* (e.g., ‘five percent’ ${-}{>}$ ‘0.05’, ‘0.05/5’ ${-}{>}$ ‘0.01’). *ngram()* extracts a definable number of words occurring before and/or after a word within a list of sentences (${\pm }$n-gram bag of words). The presence of multiple search patterns can be checked with *which.term()*. The output is either a binary hit vector for each search pattern or a vector of detected search patterns. The functions *grep2()* and *strsplit2()* are useful extensions of the basic R functions *grep()* and *strsplit()*. *grep2()* enables the identification and extraction of text using multiple search patterns linked with a logical AND. Compared to *strsplit()*, which deletes the search pattern when splitting text into pieces, *strsplit2()* allows to preserve the search pattern in the output by supporting splits before or after the recognized pattern.

**Table 1:** General text processing functions implemented in JATSdecoder and their functionality.

Function	Functionality
letter.convert()	Hexadecimal and HTML to UTF-8 conversion, CERMINE specific error correction
text2sentences()	Converts text string to vector of sentences
text2num()	Converts different representations of numbers to a digit representation
which.term()	Performs search task for multiple patterns and returns hit vector
ngram()	Extracts ±n-gram bag of words aroud a search term hit
strsplit2()	Splits text before, after, or at search pattern
grep2()	Enables search tasks on multiple search patterns connected with a logical AND

The *study.character* module bundles multiple text selection and manipulation tasks for specific contents of the list created by *JATSdecoder*. It extracts important study features such as the number of studies reported, the statistical methods applied, reported $\alpha$-level and power, correction procedures for multiple testing, assumptions mentioned, the statistical results reported, analytical software solution used, and whether the results include an analysis of interacting covariates, mediation and/or moderation effects. All functions use sophisticated, expert-guided heuristics for text extraction and manipulation, developed with great effort and domain expertise. One advantage of the time-intensive development of efficient rules is the robust recognition of a wide range of linguistic and technical representations of the targeted features, as well as a clear assignment of the causes of incorrect extractions. A functional limitation of most *study.character* functions is that they can only handle English content.

In general, *study.character* attempts to split a document into four sections (Introduction, Methods, Results, Discussion). The text of the introduction, which explains the theory and describes other work and results, and the discussion section, which contains implications, limitations, and suggestions for future procedures, can easily lead to false-positive extractions of actually realized study features. This also applies to the information in the bibliography. Therefore, mostly only the methods and results sections and captions are processed to extract the study characteristics from an article.

It has been demonstrated that *study.character*’s function *get.stats()* outperforms the program *statcheck*^17^ in extracting and recalculating p-values of statistical results reported within an article in both PDF and XML format^18^. Here, *study.character*’s functions to extract the statistical methods applied, statistical software used, number of studies per article, reported $\alpha$-level and power, test direction, correction method for multiple testing, and mentioned assumptions are evaluated using manually coded data of the study characteristics.

### Description of the extraction heuristics

A brief description of the targeted study feature and the implemented extraction heuristic of each function is given in the following section. Minor uniformization tasks are not listed, but can be traced using the source code of each function. The text processing and feature extraction are implemented with basic R functions (e.g., *grep()*, *gsub()*, *strsplit()*) and *JATSdecoder*’s text processing solutions, which are also based on these basic functions. A main feature of these functions is that they can be used with regular expressions, which makes them very powerful if used wisely. The *grep()* function performs search queries, *gsub()* finds and replaces text. Using *strsplit()*, text input can be split into a vector at text locations that match a search pattern. The search pattern itself is removed.

#### Statistical method

To draw contextual conclusions, researchers use various statistical methods and procedures to process and summarize their data. Although any descriptive as well as inferential method can be considered a statistical method, the focus here is on inferential methods. Inferential methods are based on either simple or more complex models, which also allow differing depths of data analysis and inference. Some of these methods are widespread in the literature (e.g., t-test, correlation, ANOVA, multiple regression), while other techniques are rarely used.

The function *get.method()* extracts the statistical methods mentioned in the input text. It detects sentences containing a statistical method with a list of search terms that most commonly used procedures share as an identifier (e.g., test, correlation, regression, ANOVA, method, theorem, interval, algorithm, etc.). After lowerization, up to seven preceding words with the identified search term at the end are extracted with *ngram()* and further cleaned up with an iteratively generated list of redundant words (e.g., prepositions, verbs). Users can expand the possible result space by passing additive search words to the ‘*add*’ argument of *get.method()*. The current heuristic enables the extraction of new, still unknown procedures (e.g., ‘*JATSdecoder* algorithm’), if their name ends with one of the prespecified or user-adjusted search terms. Simple descriptive measures (e.g., mean, standard deviation, proportion) are not extracted, because they are overly common and therefore do not differentiate well. Methods with a specifying term after the search term (e.g., ‘test for homogeneity of variances’) cannot be identified by *get.method()* yet.

#### $\alpha$-level

Theoretically, any frequentist decision process requires an a-priori set significance criterion, the $\alpha$-level or type-1 error probability. The type-1 or $\alpha$-error is the probability of rejecting a correct null hypothesis. Because it has become a widespread standard to work with an $\alpha$-level of 0.05, it is often not explicitly stated in practice. Among many synonyms (e.g., ‘alpha level’, ‘level of significance’, ‘significance threshold’, ‘significance criterion’) and made up terms (e.g., ‘level of confidence’, ‘level of probability’), it may be reported as critical p-value (e.g., ‘p-values $<0.05$ are considered significant’) and/or with a verbal operator (e.g., ‘the $\alpha$-error was set to 0.05’), making it difficult to detect and extract reliably. In addition, the $\alpha$-level may be reported with a value, that has been corrected for multiple testing, which does not lower the nominal $\alpha$-level. Another indirect but clearly identifiable report of an $\alpha$-error probability is the use of 1-$\alpha$ confidence intervals.

The text of the method and result sections/s, as well as the figure and table captions, are passed to *get.alpha.error()*. Prior to the numerical extraction of the reported $\alpha$-level/s, several unification tasks are performed on synonymously used terms for $\alpha$-errors and reporting styles. Levels of different p-values that are coded with asterisks are not considered $\alpha$-levels. When a corrected $\alpha$ is reported by a fraction that also contains the nominal value (e.g., ‘$\alpha =0.05/4$’) both values are returned (0.05 and 0.0125). The argument ‘*p2alpha*’ is activated by default to increase the detection rate. This option allows extraction of p-values expressing $\alpha$-levels (e.g., ‘Results with p-values < 0.05 are considered significant.’). The final output is a list distinguishing between detected nominal, corrected $\alpha$-level/s and extractions from 1-$\alpha$ confidence intervals. Since some articles report multiple $\alpha$-levels, all detected values are max- and minimized to facilitate further processing.

#### Correction for multiple testing

The nominal $\alpha$-level refers to a single test situation. When multiple tests are performed with the same $\alpha$-level, the probability of obtaining at least one significant result increases with each test and always exceeds $\alpha$. There are several correction procedures to control the inflation of the $\alpha$-error or false discovery rate, when running multiple tests on the same data.

A two-step search task is performed for the text of the methods and results section/s, as well as figure and table captions by *get.multiple.comparison()*. Sentences containing any of the search terms ‘adjust’, ‘correct’, ‘post-hoc’ or ‘multiple’ are further inspected for twelve author names (e.g., ‘Benjamini’, ‘Bonferroni’) that refer to correction procedures, as well as four specific procedures (e.g., ‘family-wise error rate’, ‘false discovery rate’) that correct for multiple testing (see Online Appendix A for the full list of specified search terms). The output is a vector with all identified authors of correction methods. Common spelling errors (e.g., ‘Bonfferoni’ instead of ‘Bonferroni’) are also detected, but returned with the correct name.

#### Test power

The concept of power describes the probability of correctly rejecting a false null hypothesis given a theoretical (a-priori) or empirical (post-hoc) effect. It can be used to estimate an optimal sample size (a-priori) or as a descriptive post-hoc measure.

*get.power()* extracts the reported aimed and achieved power value/s that are reported in the full text of the document. Since the term power is used in different contexts, sentences containing certain terms are omitted (e.g., volts, amps, hz). To reduce the likelihood of false positives, detected values that fall outside the valid power range ([0; 1]) are omitted. *get.power()* unifies some synonyms of power (e.g., $1-\beta$) and extracts the corresponding value/s if they fall within the range of 0–1. When $\beta$-errors are reported instead of power values, they are converted to power values by replacing $\beta$ with $1-\beta$.

#### Interaction effects

Analyses with more than one independent variable can be conducted with or without an interaction effect of the covariates. The term interaction effect refers to any type of interplay of two or more covariates that have dynamic effects on an outcome. In most research settings, the analysis of interactions is of great interest, as it may represent the central research hypothesis or lead to restrictions and/or reinforcement for the hypothesis/theory being tested. In addition to statistical models that explicitly include an interaction effect, mediation- and moderation analyses focus on dynamic effects of covariates on an outcome.

*has.interaction()* searches the lowerized text of the methods and results section/s for specific search patterns that relate to an interaction effect. To avoid false positive hits when analyzing articles dealing with interactions of organisms instead of variables, sentences containing specific search terms (e.g., social, child, mother, baby, cell) are removed. The output distinguishes between an identified interaction, mediator and/or moderator effect.

#### Test direction

Most research is based on theories that allow a prediction about the direction of the effect under study. Besides several procedures, that do not allow a direct conclusion about the direction of an observed effect (e.g., $\chi ^2$-Test, ANOVA), others can be applied to test directed hypotheses (e.g., t-test). Adjusting an undirected test to a directed test increases its power, if the sample and effect size are held constant, and the effect is present in the predicted direction.

Sentences containing a statistical result or one of several search terms (e.g., ‘test’, ‘hypothesis’) are searched by *get.test.direction()* for synonyms of one- and two-sided testing and hypothesis (e.g., directed test, undirected hypothesis). To avoid false positives for one-sidedness, sentences containing certain reference words (e.g., paper, page, pathway) are excluded and detected values less than one are omitted.

#### Outlier definition

Since many popular statistical measures are sensitive to extreme values (e.g., mean, variance, regression coefficients), their empirical values may not be appropriate to describe a sample. In practice, there are two popular techniques to deal with extreme values and still compute the desired statistic. Simple exclusion of outliers reduces the sample size and test power, while adjustments towards the mean preserve the original sample size. Both procedures can, of course, distort the conclusions drawn from the data because the uncertainty (variance) is artificially reduced. It is difficult to justify why valid extreme values are manipulated or removed to calculate a particular measure rather than choosing an appropriate measure (e.g., median, interquartile range). On the other hand, outliers may indicate measurement errors, that warrant special treatment. A popular measure for detecting outliers is the distance from the empirical mean, expressed in standard deviations.

*get.outlier.def()* identifies sentences containing a reference word of a removal process or an outlier value (e.g., outlier, extreme, remove, delete), and a number (numeric or word) followed by the term ‘standard deviation’ or ‘sd’. Verbal representations of numbers are converted to numeric values. Since very large deviations from the mean are more likely to indicate a measurement error than an outlier definition, and to minimize erroneous extractions of overly small values, the default result space of the output is limited to values between 1 and 10.

#### Statistical assumptions

Any statistical procedure/model is based on mathematical assumptions about the sampling mechanism, scaling, the one and/or multidimensional distribution of covariates and the residual noise (errors). The underlying assumptions justify the statistical properties of an estimator and a test statistic (e.g., best linear unbiased estimator, distributional properties, $\alpha$-error/p-value). There may be serious consequences for the validity of the conclusions drawn from these statistics, if the underlying assumptions are violated.

To extract the mentioned assumptions within an article, *get.assumption()* performs a dictionary search in the text of the methods and results sections. A total of 20 common assumptions related to the model adequacy, covariate structure, missing and sampling mechanisms can be identified (see Online Appendix C for the full list of specified search terms).

#### Analysis software

Statistical software solutions are a key element in modern data analysis. Some programs are specifically designed to perform certain procedures, while others focus on universality, performance, or usability.

To identify the analytic software solution mentioned in the methods and results sections, *get.software()* is used to perform a manually curated, fine-grained dictionary search of software names and their empirical representation in text. Tools for data acquisition or other data management purposes are not part of the list. However, they can be tracked down with a vector of user-defined search terms, passed to the ‘*add*’ argument. A total of 55 different software solutions can be detected in standard mode (see Online Appendix B for the complete list of specified search terms).

#### Number of reported studies

Research reports may contain single or multiple study reports. To determine the total number of studies reported in an article, the section titles and abstract text are passed to *get.n.studies()*. Enumerated studies or experiments are identified, and the highest value is returned. The function returns ‘1’ if no numbering of the studies is identified.

## Methods

To evaluate the extraction capabilities of *study.character*, a manually coded dataset serves as reference data. The statistical methods used, the number of studies reported, and the software solutions used were coded by Blanca et al.^7^ and provided to the author. All articles were manually rescanned for those study characteristics that are extracted by *study.character* but were not part of the original dataset.

The collection of articles by Blanca et al.^7^ consists of 288 empirical studies published in eight psychological journals (British Journal of Clinical Psychology, British Journal of Educational Psychology, Developmental Psychology, European Journal of Social Psychology, Health Psychology, Journal of Experimental Psychology-Applied, Psicothema, Psychological Research) between 2016 and 2017.

The absolute frequencies of the identified statistical procedures used in the main analysis by Blanca et al.^7^ are contrasted with those of *study.character*. The manually created categories of the statistical methods from Blanca et al.^7^ are compared to the uncategorized statistical methods extracted using *study.character*. The search tasks for counting the frequency of articles using a specific category of procedures are implemented with regular expressions. An exploratory view of the entire result space of *get.method()* is displayed in a word cloud.

To explore the correct/false positive/negative detections by *study.character* all other extracted features are compared to the manually recoded data. A correct positive (CP) detection refers to an exact match to a manually coded feature within an article. A false positive (FP) refers to an extraction that is not part of the manually coded data. Articles that do not contain a feature and for which no feature has been detected are referred to as a correct negative (CN). Finally, a false negative (FN) refers to a feature that was not detected but was manually identified.

If a target feature is identified multiple times in an article, *study.character* will output this feature once. Therefore, the evaluation of the detection rates is carried out at the article level. Since most of the features focused on here can potentially have multiple values per article, the extractions may be fully or partially correct. This can be illustrated by the example of the extraction of the $\alpha$-level. If the manual coding revealed the use of a 5% and a 10% $\alpha$-level and *study.character* identifies the 5% and an unreported 1%, this is counted to be 1 correct positive, 1 false negative and 1 false positive for this article. It follows, that the number of correct (CP+CN) and total decisions (CP+FN+CN+FP) may be larger than the total number of articles analyzed.

Global descriptive quality measures (sensitivity, specificity, accuracy) are reported for every extracted feature.

Sensitivity refers to the proportion of correctly detected features within all features present (CP+FN).

1 $$\begin{aligned} sensitivity=CP/(CP+FN) \end{aligned}$$

Specificity refers to the proportion of correct non-detections within all articles that do not contain the searched pattern (CN+FP).

2 $$\begin{aligned} specificity=CN/(CN+FP) \end{aligned}$$

Finally, accuracy is the proportion of correct detections (CP+CN) within all existing features and non-existing features (CP+FN+CN+FP).

3 $$\begin{aligned} accuracy=(CP+CN)/(CP+FN+CN+FP) \end{aligned}$$

Absolute frequency tables of manual and automatic detections are presented for each characteristic, and a causal association of the deviations that occurred is provided.

## Data, input fomats, PDF conversion software, hardware

The 288 articles in the raw data provided by Blanca et al.^7^ were manually downloaded as PDF files. The PDF files were converted to NISO-JATS encoded XML using the open-source software *CERMINE*^14^, before being processed with *study.character*. Since the compilation with *CERMINE* can lead to various errors (text sectioning/structuring, non-conversion of special characters), this can be considered as a rough test condition for the evaluated functions. All processes are performed with a Dell 4-core processor running with Linux Ubuntu 20.04.1 LTS and the open-source software R 4.0^15^. To enable multicore processing, the R package *future.apply*^19^ is used. The word cloud of the identified methods is drawn using the *wordcloud*^20^ package.

## Results

The extraction properties and causes of deviations from the manually coded study features are given in the following section for each function. A total of 287 articles are included in the analyses, as the Blanca et al.^7^ data contain one article twice.

It should be noted that the extractions of statistical methods and software solutions from Blanca et al.^7^ are not directly comparable to the output of *study.character* as they coded the statistical methods used in the main analyses (rather than each mention) and that are explicitly reported to be used for these main analyses.

### Statistical methods

**Figure 1:**
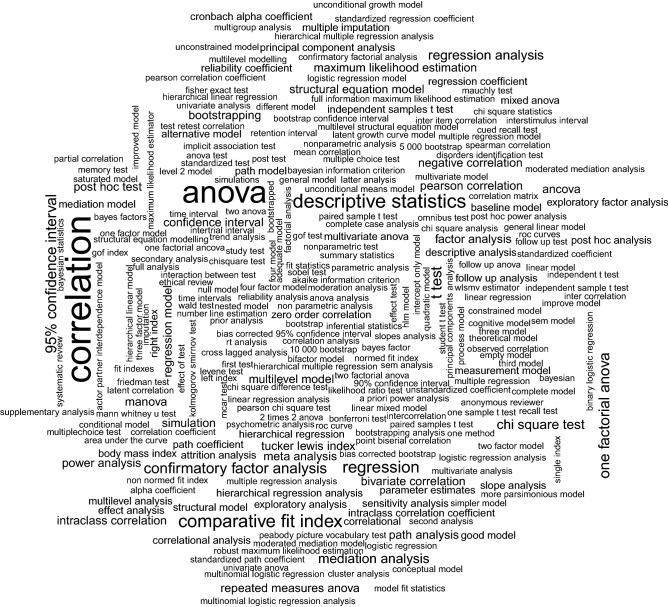
Word cloud of the extracted statistical methods by *study.character*.

An insight into the overall result space of the statistical methods extracted by *study.character* is given in Fig. 1, where the frequency table of the extractions is shown as a word cloud. Bigger words indicate higher frequencies. It is obvious, that correlation analysis and ANOVA are the most frequently mentioned methods in this article selection.

In order to compare the extractions of *get.method()* with the extractions of the main analysis procedure of Blanca et al.^7^ the absolute frequencies of the detected studies using a specific class of methods are listed in Table 2. The regular expressions listed are used as search terms to count the hits of *get.method()* per categorized method.

Because Blanca et al.^7^ coded the statistical method used in the main analysis (all methods reported in preliminary analyses or manipulation checks, footnotes, or in the participants or measures section, were not coded), most methods are more commonly identified by *get.method()*. Two rare categories cannot be identified at all with the search terms used (‘correlation comparison test’, ‘multilevel logistic regression’ $^{\wedge }$).

**Table 2:** Number of articles with mentions of specific statistical methods extracted with *study.character* and frequency of main analytical methods reported in Blanca et al.’s Table 4.

Statistical method	Search term	Blanca et al.	get.method()	$$\Delta $$
Descriptive statistics (M; SD; OR; RR; percentages; etc.)	‘Descriptive statistics|descriptive analysis|descriptives’	15	63	48
Distribution fitting	‘Distributional analysis|distribution analysis|distribution fitting’	3	1	-2
Inter-rater agreement (Kappa; Intraclass correlation coefficient)	‘Inter rater reliability|kappa|intraclass correlation|intra class correlation’	3	30	27
Pearson’s correlation coefficient	‘Pearson correlation|pearson product|product moment|zero order correlation| $$^{\wedge }$$correlation$’	54	138	84
Pearson’s correlation/regression coefficient comparison test	‘Correlation comparison|coefficient comparison’	3	0	-3
Spearman’s correlation coefficient	‘Spearman corr|spearman brown|spearmancoef |spearman rank|spearman rho’	8	12	4
Other nominal/ordinal correlation measures (gamma; Cramer’s V; Somers’ d; contingency coefficient)	‘Gamma|cramer v|somer d|contingency table analysis|contingency coef’	4	3	-1
Pearson’s Chi-square	‘$$^{\wedge }$$chi square|[$$^{\wedge }$$d] chi square’	27	52	25
Other test of contingency tables and proportion comparison (Fisher; McNemar tests; Cochran’s Q; z statistic)	‘Fisher exact|fisher z|mcnemar|cochran q|z statistic’	5	9	4
Mann-Whitney U test	‘Mann whitney|mannwhitney|u test’	5	7	2
Wilcoxon signed-rank test	‘Wilcoxon|signed rank’	4	4	0
Kruskal-Wallis test	‘Kruskal |wallis ’	5	5	0
Friedman test	‘Friedman test’	5	7	2
One sample t-test	‘One sample t test|single sample t test’	11	7	-4
Independent t-test	‘$$^{\wedge }$$t test$ |[$$^{\wedge }$$n][$$^{\wedge }$$e] t test|independent t test|two sample t test’	45	62	17
Paired t-test	‘Paired samples t test|paired sample t test|paired t test’	20	18	-2
ANOVA	‘Anova| $$^{\wedge }$$anova’	138	156	18
ANCOVA	‘Ancova| $$^{\wedge }$$ancova’	15	15	0
MANOVA/MANCOVA	‘Manova|mancova’	13	19	6
Regression analysis	‘Regression’	82	127	45
Multilevel regression	‘Multilevel.*?regression|hierarchic.*?regression|mixed.*?regression’	25	31	6
Multivariate regression	‘Multivariate.*?regres|multiple*?regres’	1	3	2
Poisson regression	‘Poisson regression’	1	1	0

Statistical method	Search term	Blanca et al.	get.method()	$\Delta$
Logistic regression	‘Log.*?regression’	15	28	13
Multilevel logistic regression	‘Multilevel logistic|logistic multilevel’	5	0	-5
Multinomial/ordinal regression	‘Multinom.*?regres|ordin.*?regres’	9	10	1
Generalized estimation equation for ordinal data	‘Gee|generalized estimation equation’	1	1	0
Path analysis	‘Path analysis|path model|path coefficient|path estimate|structural equation’	46	53	7
Multilevel SEM	‘Multilevel structural equation’	6	4	-2
Growth curve modeling	‘Growth curve|growth model’	10	11	1
Multilevel growth curve modeling	‘Multilevel growth|multigroup.*?growth’	2	1	-1
Confirmatory factor analysis (CFA; SEM)	‘Confirmatory factor’	21	35	14
Exploratory factor analysis (EFA)	‘Exploratory factor’	13	14	1
Cronbach’s $\alpha$	‘Cronbach alpha|reliability coeff|cronbach coeff’	17	25	8
McDonald’s omega	‘Mcdonald|mc donald|omega coef|omega estimate’	4	3	-1
Test’retest reliability	‘Test retest reliability|test retest corr’	3	4	1
Convergent/Discriminant validity indexes (AVE; MSV)	‘Convergent validity|discriminant validity|[$^{\wedge }$a-z]ave $|{^{\wedge }}$ave |msv’	2	2	0
Sensitivity and specificity measures	‘Sensitivity|specificity|specifity’	2	14	12
Item analysis: classical test theory	‘Item analysis|items analysis’	2	4	2
Item analysis: item response theory (item calibration; DIF)	‘Item response analysis|dif analysis’	2	2	0
Cluster analysis	‘Cluster analysis’	3	4	1
ROC curve analysis	‘Roc curve|receiver operating’	7	7	0
Markov models	‘Markov|marcov’	1	1	0

The large differences in most of the identified methods (e.g., descriptive statistics, correlation, $\chi ^2$-statistics) are due to the different inclusion criteria (each mentioned method vs. method of main analysis). In addition, using of ‘regression’ as a search term in the output of *get.method()* also results in hits when more complex regression models were found (e.g., multilevel or multivariate regression), whereas Blanca et al.^7^ consider simple regression models and more specific regression models to be disjoint.

### Sensitivity, specificity, accuracy

Table 3 shows the sensitivity, specificity, and accuracy measures for *study.character*’s extractions based on the manually coded data. Most of the extractions work very accurately and can replace a manual coding.

Except for the $\alpha$-level detection with ‘*p2alpha*’ activated, all extractions have low false positive rates. In default mode, the empirical sensitivity of all extractions is above 0.8, the specificity above 0.9. Since there are usually very few false positive extractions, five specificity measures reach 1.

Accuracy is lowest for $\alpha$-level detection (0.86 with ‘p2alpha’ deactivated, 0.9 in default mode) and statistical assumption extraction (0.9). The accuracy of all other extractions is above 0.9. The binarized outputs for the extracted interaction and the stated assumptions have higher accuracy than the raw extractions.

**Table 3:** Sensitivity, specificity and accuracy of *study.character*’s extractions.

Feature	CP	CN	FP	FN	$\Sigma$	Sensitivity	Specifity	Accuracy
$\alpha$-level from CI	105	169	0	21	295	0.83	1	0.93
Max of $\alpha$-level and CI (p2alpha=FALSE)	125	122	1	40	288	0.76	0.99	0.86
Max of $\alpha$-level and CI (p2alpha=TRUE)*	137	120	2	28	287	0.83	0.98	0.90
Power	61	242	2	12	317	0.84	0.99	0.96
Correction for multiple testing	65	230	0	2	297	0.97	1	0.99
Outlier removal	24	262	0	1	287	0.96	1	1
Test direction	33	252	1	1	287	0.97	1	0.99
Interaction/mediator/moderator	242	87	10	22	361	0.92	0.90	0.91
Interaction (binary)	192	87	2	6	287	0.97	0.98	0.97
Assumptions	79	215	19	12	325	0.87	0.92	0.90
Assumptions (binary)	64	215	5	3	287	0.96	0.98	0.97
Software	245	105	0	8	358	0.97	1	0.98
n studies	283	0	4	4	291	0.99	0	0.97

Detections may sum up to values $>N=287$ as results may be multidimensional and false positives are included.

*Default setting of study.character.

### $\alpha$-level

Although most of the studies examined make use of inferential statistics, only 78 (27%) explicitly report an $\alpha$-level. In all cases, where no $\alpha$-level is reported, the standard of $\alpha =5\%$ is applied, but not considered an extractable feature. Since some studies report the use of multiple $\alpha$-levels, the total number of detected and undetected $\alpha$-levels exceeds the number of articles. Eight articles report the use of a 90% confidence interval and a 95% confidence interval.

The absolute frequency of $\alpha$-levels extracted from 1-$\alpha$ confidence intervals by *study.character* and the manual analysis are shown in Table 4. *study.character* correctly extracts the $\alpha$-value in 105 out of 126 (83%) confidence interval reports in 97 out of 118 (82%) articles. No false positive extraction is observed. Seven non-detections by *study.character* are due to *CERMINE* specific conversion errors of figure captions, 11 to the non-processing of column names and content of tables. Two reports of confidence intervals cannot be recognized due to unusual wording (‘95% confidence area’, ‘confidence intervals set to 0.95’), one due to a report in the unprocessed discussion section.

**Table 4:** Absolute frequencies of detected $\alpha$-level from 1-$\alpha$ confidence intervals.

$\alpha$-level	study.character	Manual coding	False positive	False negative
0.01	1	1	0	0
0.02	1	1	0	0
0.05	89	109	0	20
0.1	14	15	0	1
Sum	105	126	0	21

The corrected $\alpha$-level cannot be well distinguished from an uncorrected $\alpha$-value. Only one out of eight corrected $\alpha$-levels is correctly labeled and extracted by *study.character*, one is a false positive detection of a nominal $\alpha$. The extracted nominal $\alpha$-level contains three of the manually extracted corrected $\alpha$-values.

For simplicity, the extracted nominal and corrected $\alpha$-levels are merged with the extraction from the confidence intervals and reduced to their maximum value, which corresponds to the nominal $\alpha$-level. Table 5 shows the frequency distribution of the extracted maximum $\alpha$-level with the deactivated conversion of p- to $\alpha$-values and the default setting.

**Table 5:** Distribution of extracted maximum $\alpha$-level with option ‘p2alpha’ deactivated and in default mode (numbers in brackets).

$\alpha$-level	study.character	Manual coding	False positive	False negative
0.003	0 (0)	1	0 (0)	1 (1)
0.01	2 (3)	2	1 (2)	1 (1)
0.0125	1 (1)	1	0 (0)	0 (0)
0.017	1 (1)	1	0 (0)	0 (0)
0.02	1 (1)	1	0 (0)	0 (0)
0.05	107 (119)	144	0 (0)	37 (25)
0.1	14 (14)	15	0 (0)	1 (1)
Sum	126 (139)	165	1 (2)	40 (28)

The conversion procedure of p-values increases the accuracy of $\alpha$-level extraction, but brings one additional false positive extraction, which is caused by a statistical test result reported with a standard threshold of p-values ($p<0.01$). Thus, enabling the conversion of p- to $\alpha$-values slightly increases the false positive rate of explicitly reported $\alpha$-levels, especially for rather rarely applied levels (0.1, 0.01 and 0.001).

### Test power

Since test power can be reported as both a-priori and a-posteriori results, some articles contain multiple power values. The absolute distribution of categorized power values found by *study.character* and manual coding is shown in Table 6. The evaluation of the categorized power values differs from the results in Table 3 because here, four unrecognized values in articles with several power values of the same category are evaluated as fully correct. There are two false-positive extractions caused by a poorly compiled table and a citation of Cohen’s recommendation to plan studies with at least 80% power^21^. Both errors occur in documents that contain other correctly extracted power values. Overall, 61 of 73 (84%) manually coded and categorized power values are correctly extracted in 42 of 45 (93%) articles. Nine of the 12 unrecognized reports of power follow a text structure that is still unmanageable (e.g., ‘The final sample size ensured sufficient power (i.e., 0.99)’, ‘The statistical power was very high (0.99)’). This also applies to the specification of a power interval (‘with a power ranging between 0.80 and 0.90’). Here, only the first value (0.8) was extracted and considered a correct positive, while the second limit of the interval is missing and considered a false negative. One non-detection is caused by an uncompiled and unimputated Greek letter $\beta$. In addition, one erroneous report of a power value of 80 is not extracted by *study.character*, because it falls outside the defined result space [0; 1]. Further, one power value reported in an unprocessed figure caption is not detected.

**Table 6:** Absolute frequencies of extracted categorized test power.

Power	study.character	Manual coding	False positive	False negative
(0,0.2]	5	5	0	0
(0.2,0.5]	3	3	1	1
(0.5,0.79]	12	12	0	0
(0.79,0.8]	25	24	1	0
(0.8,0.9]	6	8	0	2
(0.9,1]	12	20	0	5
$>1$	0	1	0	0
Sum	63	73	2	8

### Correction for multiple testing

Table 7 shows the absolute frequency of detected correction method for multiple testing by *study.character* and the manual coding. Within the collection of articles analyzed, ten of 15 detectable authors/correction methods for multiple testing are identified by *study.character* without a false positive. There are two non-identifications. One article reports a p-value correction, but not the specific method. In another article, the reported use of a ‘Bonferroni Test’ is not detected as a correction procedure, because it is not mentioned that something is corrected/adjusted with it.

**Table 7:** Absolute frequencies of authors of multiple test correction procedures.

Author	study.character	Manual coding	False positive	False negative
Bonferroni	38	39	0	1
Tukey	9	9	0	0
Holm	6	6	0	0
Fisher LSD	3	3	0	0
Hochberg	3	3	0	0
Scheffé	2	2	0	0
Benjamini	1	1	0	0
Duncan	1	1	0	0
Keuls	1	1	0	0
Newman	1	1	0	0
not specified	0	1	0	1
Sum	65	67	0	2

### Interaction effects

The distinction between moderation, mediation, and interaction effects works in 242 out of 264 mentions (92%) (see Table 3). Table 8 shows the frequency of extracted type of interaction effect by *study.character* and the manual coding.

**Table 8:** Absolute frequencies of specific interaction effects.

	study.character	Manual coding	False positive	False negative
Interaction	151	158	2	9
Mediator	68	70	3	5
Moderator	33	36	5	8
Sum	252	264	10	22

Overall, 22 specific mentions are not recognized in 20 articles, and 10 false positive hits occurred in 10 articles. Unrecognized mentions are mostly due to reports within the non-scanned abstract, introduction, discussion, or section headings as well as simple but badly handled sentences (e.g., ‘The model also included the interactions between A and each predictor variable.’ or ‘We tested the effect of A and B, along with their interaction.’). The false positive extractions are mainly observed in studies that infer moderating/mediating effects of covariates but do not perform an explicit moderator/mediator analysis. In two articles examining mother-infant interaction and quality of interactions among peers, exclusion of target sentences fails for the term ‘interaction’ and causes false positive extractions.

The presence of an interaction effect can be localized very well with the binarized output (no detection vs. any detection of interaction). The presence of at least one type of interaction analysis is correctly detected in 192 of 198 (97%) articles. In total, six articles analyzing an interaction effect of variables are not identified, two detections are false positives.

### Outlier definition

The distribution of detected outlier definitions is shown in Table 9. Twenty-four out of 25 (96%) outlier definitions, expressed by standard deviations, are correctly extracted. One report of an outlier removal is not detected due to an non-compiled special character (‘±’). This error does not occur when parsing the original sentence (‘Twenty-nine additional infants were excluded for following reasons: ..., extreme looking times (±2 SD) ...’) Because only removal processes reported with standard deviations are targeted, one outlier removal based on an interquartile range of 1.5 is not part of this analysis.

**Table 9:** Absolute frequencies of extracted outlier definition expressed in standard deviations (SD).

SD	study.character	Manual coding	False positive	False negative
2	4	5	0	1
2.5	8	8	0	0
3	12	12	0	0
Sum	24	25	0	1

### Test direction

The absolute distribution of detected test sidedness by *study.character* and the manual coding is shown in Table 10. Thirty-three of 34 (97%) reports of a reported test direction are correctly extracted. One false positive hit is observed in an article dealing with ‘one-sided aggression’ One report of a two tailed test setting is not detected in a sentence about power considerations (‘...a difference of $d=0.4$ ($\eta ^2=0.04$; two tails, $\alpha =0.05$)...’), because ‘two tails’ is not defined as inclusion pattern.

**Table 10:** Absolute detections of test direction/s.

Test direction/s	study.character	Manual coding	False positive	False negative
One and two sided	2	2	0	0
One sided	6	5	1	0
Two sided	26	27	0	1
Sum	34	34	1	1

### Statistical assumptions

Seventy-nine of 91 manually coded assumptions (87%) are extracted correctly (see Table 3). Extraction of specific assumptions results in more false positive (19) than false negative (12) detections. Both, the false positive and negative detections mainly concern the very general assumptions of normally distributed variables, independence of measurements and linearity of relationships. More specific assumptions are extracted very accurately. Four manually extracted assumptions are missed because they are not part of the result space of *get.assumption()* (homogeneity of covariance matrices, sampling adequacy, non proportionality) or are too unspecific (homogeneity). For one article containing the manually coded assumption of non-proportionality, *study.character* outputs the proportional hazards and proportional odds assumptions, which is appropriate. The absolute frequency distribution of the extracted assumptions by *study.character* and the manual coding is shown in Table 11.

**Table 11:** Absolute frequencies of detected statistical assumptions.

Assumption	study.character	Manual coding	False positive	False negative
Normal distribution	23	19	6	2
Sphericity	18	18	1	1
Missing at random	14	15	0	1
Missing completely at random	8	8	0	0
Independency	7	5	3	1
Linearity	7	5	4	2
Multivariate normal	5	5	0	0
Homogeneity of variances	4	2	2	0
Multicollinearity	4	5	0	1
Equal variances	3	2	1	0
Homoscedasticity	2	2	0	0
Autocorrelation	1	1	0	0
Proportional hazards	1	0	1	0
Proportional odds	1	0	1	0
Homogeneity	0	1	0	1
Homogeneity of covariance matrices	0	1	0	1
Nonproportionality	0	1	0	1
Sampling adequacy	0	1	0	1
Sum	98	91	19	12

### Analysis software

Compared to the manually recoded software mentions, the dictionary search tasks work very accurately. Eight software mentions are missed, no false positive extraction is observed. In total, *get.software()* identifies 245 usages of 23 different software solutions in 181 articles. This is significantly more than reported by Blanca et al.^7^ (180 uses of 13 software programs in 155 articles). The absolute frequencies of studies explicitly reporting the use of a statistical software to perform the main analysis coded by Blanca et al.^7^ and all extracted software mentions by the *get.software()* function of *study.character* are listed in Table 12.

**Table 12:** Absolute frequencies of detected software solutions by *study.character*, the manually coded data, and the explicitly stated software solution used for the main analyis reported in Blanca et al.’s Table 7.

Software	study. character	Manual coding	Blanca et al.
SPSS	71	71	52
Mplus	45	45	44
PROCESS MACRO	25	25	26
AMOS	18	18	19
G*Power	14	16	0
MATLAB	13	13	0
Stata	11	11	8
SAS	10	10	6
R	9	9	7
HLM	5	5	0
EQS	3	3	3
JASP	3	3	0
Excel	3	3	0
LISREL	2	2	2
FACTOR	2	2	2
Python	2	2	0
Sum	238	236	169
Psychtoolbox	2	2	0
AcqKnowledge	2	2	0
MLwiN	1	1	1
Warp PLS	1	1	0
Statistica	1	1	0
Praat	1	1	0
ImageJ	1	1	0
TimeStudio	1	0	0
Rmediation Webapp	1	0	0
Omega	1	0	0
ConQuest 2.0	1	0	0
ChiSquareDif	1	0	0
ASY DIF	1	0	0
HTLM	0	0	6
other	0	0	4
Sum	15	9	11

A total of six discrepancies to the recoded data of software mentions are due to extraction errors of *study.character* involving non-captures of G*Power (2), the tool PROCESS MACRO (2), MPlus (1) and Amos (1). Compared to the analysis of Blanca et al.^7^, with the exception of AMOS and SPSS PROCESS MACRO, all other software solutions are identified more frequently or as frequently by *study.character*. A comparison with the manual coding shows that most of the discrepancies (47) are due to different inclusion criteria (mentioned vs. explicitly stated software for the main analysis). Since G*Power is never used to perform the main calculations of an analysis, it is not extracted by Blanca et al.^7^. All mentions of Matlab (13) extracted with *get.software()* are not included in the data of Blanca et al.^7^, since it was only indicated that it was used to design the computerized experiment, with no explicit indication of its use as analysis software. In eight cases, neither Blanca et al.^7^ nor *study.character* extracted the specifications of comparatively rarely used software solutions (e.g., ConQuest 2.0, ASY-DIF), because they were outside their area of interest, or results space. Nevertheless, these software solutions could be easily detected by *get.software()* by adding these names to the ‘add’ argument. Some tools such as Omega, ChiSquareDif or Excel extracted by *get.software()* were outside the domain of interest of Blanca et al.^7^.

### Number of studies reported per paper

In 283 articles (98.6%), the number of contained studies is correctly extracted by *study.character* (see Table 3). Since a numeric value is returned in each case, the function has no specificity. The output can only be right or wrong, and never false negative. Compared to the manually recoded data, there are four false detections by *study.character*. All are due to missing section names in the compiled XML file. In one case, only some relevant section names were not compiled by *CERMINE*, resulting in an output of ‘2’ instead of ‘3’ studies. In three cases, the missing section names result in the default output of ‘1’ study. The full distribution of the number of reported studies per paper extracted by *study.character*, the manual recoding and Blanca’s analysis are shown in Table 13.

**Table 13:** Absolute frequencies of extracted number of studies per paper by *study.character*, the manually coded data and Blanca et al.’s Table 2.

N studies:	1	2	3	4	5	6
study.character	205	36	24	15	6	1
Manual coding	202	37	26	15	6	1
Blanca et al.	200	39	29	15	4	0

In contrast to the manually coded data of Blanca et al.^7^ (Table 2 in the original article), there are seven deviations from the recoded number of studies per article. Some differences can be explained by different coding approaches. Two control tasks in one study were treated as two individual studies by Blanca et al.^7^, while *study.character* does not consider them as a study. One study with two sub-studies in a single study was coded as two studies by Blanca et al.^7^. No error assignment can be made for the remaining five discrepancies. These include an article with six studies coded as an article with three studies by Blanca et al.^7^.

## Discussion

The accuracy analysis of the study feature extraction presented here contributes to the establishment of *JATSdecoder* as a useful tool for science research. In addition to the extraction algorithms for statistical results that have already been evaluated^18^, key methodological features of studies can now be extracted in a rubust way, opening up a wide range of new possibilities for research on research projects.

*JATSdecoder*’s module *study.character* can facilitate meta-research and identification tasks on methodological study features of scientific articles, that are written in English. The primary focus on NISO-JATS encoded content and the purely heuristics-based approach distinguish *JATSdecoder* from most other text processing packages. Nevertheless, the implemented extraction heuristics exhibit high accuracy and can replace overly time-consuming human coding. Moreover, *study.character* facilitates the monitoring of methodological research practices in large text databases. Such analysis can be performed for one or many journals, authors, subjects, and/or disciplines when the output of *study.character* is combined with the metadata extracted by *JATSdecoder*.

Although *study.character* cannot distinguish between a statistical method and other text phrases that are not statistical methods (e.g., ‘IQ test’), its output facilitates research on research that is focused on the application and distinction of statistical methods in practice. A reduction of dimensionality by search terms enables rapid method-specific identification of studies and mapping of developments in scientific research practice.

However, some aspects should be considered in an individual or overall analysis of studies with *study.character*. Compared to Blanca et al.^7^, who extracted the main statistical method used and the explicitly stated software used to perform the main calculations, *study.character* outputs all mentioned methods and software solutions within the methods and results sections.

A key feature of *study.character* is the selection of section-specific text parts, which only works for NISO-JATS encoded XML files. The very low false positive rates indicate that the text selection and exception handling work properly. Nevertheless, applying the *study.character* functions to any unstructured plain text is possible, but may lead to more false positive extractions, if discussions and reference lists are also processed.

The evaluation was done on psychological studies only. Whether the high level of precision can be achieved in other scientific disciplines, thus allowing a general analysis of scientific procedures in other disciplines, remains to be answered by future research.

There are other interesting study features that are not yet extracted by *study.character*. For example, an identification by study design (e.g., experimental, observational), study type (e.g., randomized treatment control, placebo waitlist control), or measurement tools (e.g., questionnaire, EEG, DNA-sequencing) might be of interest in a meta-analysis. Since all of these features are high-dimensional when considered across a broad range of scientific practice, consideration should be given to developing more sophisticated natural language processing tools that can address this issue. In any case, it should be carefully investigated whether model-based text extraction tools (e.g., Named Entity Recognition) can outperform both methods. One aspect that will be complicated when using these methods is assigning the cause of incorrect extractions.

A web application enabling the analysis and selection of the extracted metadata and study characteristics of content within the PMC database is provided at: https://www.scianalyzer.com. The handling is simple and allows even inexperienced users to make use of the *JATSdecoder* extractions and perform individual analysis and search tasks. The raw data of searches with less than 20,000 results can be downloaded for further processing.

Since *JATSdecoder* is a modular software, externally developed extraction functions can be easily implemented. Collaboration on *JATSdecoder* is very welcome to further improve and extend the functionality. It can be initiated via the GitHub account: https://github.com/ingmarboeschen/JATSdecoder.

## Supplementary Information

Supplementary Information.

## Data Availability

An interactive web application for analyzing study characteristics and identifying articles linked in the PubMed Central database is accessible at: https://www.scianalyzer.com.

## Code availability

*JATSdecoder* software is freely available at: https://cran.r-project.org/package=JATSdecoder; https://github.com/ingmarboeschen/JATSdecoder. Scripts to reproduce this and other analyses performed with* JATSdecoder* are stored at: https://github.com/ingmarboeschen/JATSdecoderEvaluation.
