# Supplementary material for: Evaluation of the extraction of methodological study characteristics with JATSdecoder
Source: Sci Rep. 2023 Jan 4;13:139. doi: 10.1038/s41598-022-27085-y (PMC9813005; doi:10.1038/s41598-022-27085-y)
Supplement: Supplementary file 1 — Supplementary Information. [file 41598_2022_27085_MOESM1_ESM.pdf]

Appendix A

Table 14. Regular expressions used as search terms for the dictionary search to extract the author and procedure of correction method for multiple testing

<table>
<tr><th>correction method</th><th>regular expression</th></tr>
<tr><td><u>Author</u></td><td></td></tr>
<tr><td>Benjamini</td><td>‘Benjamini’</td></tr>
<tr><td>Bonferroni</td><td>‘Bonferroni|[Bb]onff*err*onn*i’</td></tr>
<tr><td>Boole</td><td>‘Boole[^a-z]’</td></tr>
<tr><td>Duncan</td><td>‘Duncan’</td></tr>
<tr><td>Dunnett</td><td>‘Dunnett[^a-z]|Dunn*et’</td></tr>
<tr><td>Hochberg</td><td>‘Hochberg’</td></tr>
<tr><td>Holm</td><td>‘Holm[^a-z]’</td></tr>
<tr><td>Keuls</td><td>‘Keuls’</td></tr>
<tr><td>Newman</td><td>‘Newman’</td></tr>
<tr><td>Scheffé</td><td>‘Scheff[eéè]|Schef*[eéè]|Scheff[^a-z]’</td></tr>
<tr><td>Šidák</td><td>‘[ŠS]id[aá]k’</td></tr>
<tr><td>Tukey</td><td>‘Tukey|Tuckey’</td></tr>
<tr><td></td><td></td></tr>
<tr><td><u>Procedure</u></td><td></td></tr>
<tr><td>AlphaSim</td><td>‘AlphaSim|Alpha[-]Sim’</td></tr>
<tr><td>Family-Wise Error Rate</td><td>‘[^a-z]FWER*[^a-z]|[Ff]amily[-]*[Ww]ise [Ee]rror [Rr]ate’</td></tr>
<tr><td>False Discovery Rate</td><td>‘[^A-Z]FDR[^A-Z]|[fF]alse [dD]iscovery [rR]ate’</td></tr>
<tr><td>Least Significant Difference</td><td>‘Fisher LSD|[Ll][Ss][Dd][^A-Za-z].*post[-]hoc|post[-]hoc.*[^a-zA-Z][Ll][Ss][Dd]|
LSD[^A-Z].*Fisher|Fisher[^a-z].*LSD[^a-z]|[Ll]east [Ss]ignificant [Dd]ifference’</td></tr>
</table>

Appendix B

Table 15. Regular expressions used as search terms for the dictionary search of analysis software used

<table>
<tr><th>software</th><th>regular expression</th></tr>
<tr><td>SPSS</td><td>‘SPSS|Statistical Package for the Social Sciences|PASW|Predictive Analytics Soft[Ww]are’</td></tr>
<tr><td>R</td><td>‘R Core|using R[,\\.]| in R[,\\.]|[^a-zA-Z][Rr] [Pp]roject|oftware ’*R’*[^a-zA-Z]|
[A-Za-z]R Foundation| with R[,\\.]|R Development Core|[^A-Z0-9] R \\([Vv1-4]| R [0-9]\\.[0-9]|
R[0-9]\\.[0-9]|[Ss]oftware R[^a-zA-z]|[^a-zA-Z]R [Ss]oftware|anguage R|R [Ff]oundation|
in R [Vv]ersion|with R [Vv]ersion| in R with’</td></tr>
<tr><td>SPM</td><td>‘[^A-Z]SPM[1-9]|[^A-Z]SPM.[0-9]|^SPM[1-9]|^SPM.[0-9]|
[sS]tatistical [pP]arametric [mM]apping’</td></tr>
<tr><td>Stata</td><td>‘Stata[^a-z]|STATA[^A-Z]’</td></tr>
<tr><td>SAS</td><td>‘SAS Institute|Statistical Analysis Software|Statistical Analysis System|[\\( ]SAS [Vv]ersion [1-9]|
with SAS [Vv]ersion| in SAS [1-9]|with SAS [1-9]|SAS PROC’</td></tr>
<tr><td>Statistica</td><td>‘Statistica |STATISTICA|StatSoft’</td></tr>
<tr><td>PROCESS MACRO</td><td>‘PROCESS MACRO|PROCESS *[Mm]acro|PROCESS [Mm]odul|PROCESS.*Hayes|
Hayes.*PROCESS|Process *[Mm]acro|PROCESS *[Tt]oolbox|PROCESS [a-z]* *tool’</td></tr>
<tr><td>MATLAB</td><td>‘MATLAB|Matlab’</td></tr>
<tr><td>NVivo</td><td>‘N[Vv]ivo|N Vivo’</td></tr>
<tr><td>MAXQDA</td><td>‘MAXQ[DA][AD]|MAXqda’</td></tr>
<tr><td>AcqKnowledge</td><td>‘Acq[Kk]nowledge’</td></tr>
<tr><td>AFNI</td><td>‘AFNI’</td></tr>
<tr><td>FreeSurfer</td><td>‘Free[Ss]urfer|Free Surfer’</td></tr>
<tr><td>GraphPad</td><td>‘Graph[Pp]ad|Graph Pad’</td></tr>
<tr><td>REST</td><td>‘[^A-Z]REST[^A-Z]’</td></tr>
<tr><td>ImageJ</td><td>‘ImageJ|Image J[^a-z]’</td></tr>
<tr><td>JMP</td><td>‘JMP[^a-z]’</td></tr>
<tr><td>Excel</td><td>‘Excel[^a-z]|[^A-Z]EXCEL|XLSTAT’</td></tr>
<tr><td>FMRIB</td><td>‘[Ff]MRIB|[^A-Z]FSL[^A-Z]’</td></tr>
<tr><td>G*Power</td><td>‘[^A-Za-z]G *Power|^G *Power’</td></tr>
<tr><td>PASS</td><td>‘[^A-Z]PASS[^A-Z]’</td></tr>
<tr><td>NCSS</td><td>‘[^A-Z]NCSS[^A-Z]’</td></tr>
<tr><td>Praat</td><td>‘Praat|PRAAT’</td></tr>
<tr><td>AMOS</td><td>‘AMOS|Amos[^a-z]’</td></tr>
<tr><td>LISREL</td><td>‘LISREL|Lisrel[^a-z]’</td></tr>
<tr><td>MPlus</td><td>‘[^A-Za-z][Mm][Pp][Ll][Uu][Ss]|[^A-Za-z][Mm] [Pp][Ll][Uu][Ss]’</td></tr>
<tr><td>Python</td><td>‘Python|PYTHON’</td></tr>
<tr><td>StudSize</td><td>‘StudSize’</td></tr>
<tr><td>Psychtoolbox</td><td>‘Psychtoolbox|Psych [Tt]oolbox|Psych[tT]oolbox’</td></tr>
<tr><td>PsyScope</td><td>‘Psy[Ss]cope|Psy [Ss]cope’</td></tr>
<tr><td>Systat</td><td>‘Systat|SigmaPlot|SigmaStat|SYSTAT’</td></tr>
<tr><td>MLwiN</td><td>‘MLwiN|ML[wW][Ii][nN]’</td></tr>
<tr><td>WINanalyze</td><td>‘WINanalyze|Win[-]*Analyze’</td></tr>
</table>

Continuation of table 12: Regular expressions used as search terms for the dictionary search of analysis software used

<table>
<tr><th>software</th><th>regular expression</th></tr>
<tr><td>C++</td><td>‘C[+][+]’</td></tr>
<tr><td>StatGraphics</td><td>‘Statgraphics|StatGraphics’</td></tr>
<tr><td>EQS</td><td>‘EQS[^A-Z]’</td></tr>
<tr><td>Smart PLS</td><td>‘Smart PLS|SmartPLS’</td></tr>
<tr><td>Warp PLS</td><td>‘Warp PLS|WarpPLS’</td></tr>
<tr><td>Winsteps</td><td>‘Winsteps|WinSteps|WINSTEPS’</td></tr>
<tr><td>MindWare</td><td>‘MindWare|Mindware|MINDWARE’</td></tr>
<tr><td>FACTOR</td><td>‘FACTOR|oftware Factor|Factor [Ss]oftware|Factor [1-9]\\.[0-9]|Factor [1-9][0-9]\\.[0-9]’</td></tr>
<tr><td>JASP</td><td>‘JASP’</td></tr>
<tr><td>Kubios</td><td>‘Kubios’</td></tr>
<tr><td>Neuroscan</td><td>‘Neuroscan’</td></tr>
<tr><td>DMDX</td><td>‘DMDX’</td></tr>
<tr><td>EEGLAB</td><td>‘EEGLAB’</td></tr>
<tr><td>MRICro</td><td>‘MRI[Cc]ro[^Nn]’</td></tr>
<tr><td>MRICroN</td><td>‘MRI[Cc]ro[Nn]’</td></tr>
<tr><td>Tanagra</td><td>‘Tanagra’</td></tr>
<tr><td>OptoGait</td><td>‘OptoGait’</td></tr>
<tr><td>Cartool</td><td>‘Cartool’</td></tr>
<tr><td>RDSAT</td><td>‘RDSAT’</td></tr>
<tr><td>METAWIN</td><td>‘METAWIN|META[-]*WIN’</td></tr>
<tr><td>Review Manager</td><td>‘Review Manager|REVIEW MANAGER’</td></tr>
<tr><td>HLM</td><td>‘HLM [1-9vV]|HLM[Pp]rogram|HLM [Ss]oft|[Hh]ierarchical [Ll]inear [Mm]odeling [Pp]rogram|
[Hh]ierarchical [Ll]inear [Mm]odeling [Ss]oft’</td></tr>
<tr><td>QtiPlot</td><td>‘QtiPlot’</td></tr>
<tr><td>Grapher</td><td>‘Grapher[^a-z]’</td></tr>
<tr><td>Open-MX</td><td>‘Open[-]*MX’</td></tr>
</table>

Appendix C

Table 16. Regular expressions used as search terms for the dictionary search of mentioned assumptions

<table>
<tr><th>assumption</th><th>regular expression</th></tr>
<tr><td>linearity</td><td>‘[a-z]linearity|[a-z]linear|linearity|^linear’</td></tr>
<tr><td>Gauss-Marcov assumptions</td><td>‘gauss-marcov|gauss[-]mar[ck]o[wv]|gau[sß][-]mar[ck]o[wv]|
^mar[ck]ov assumption|[^sß] mar[ck]ov assumption’</td></tr>
<tr><td>homoscedasticity</td><td>‘homoscedasticity|homo[-]*scedastic’</td></tr>
<tr><td>no autocorrelation</td><td>‘autocorrelation|auto[-]correlation|autocor|[^a-z]acf[^a-z]’</td></tr>
<tr><td>no multicollinearity</td><td>‘multicollinearity|multi[-]*coll*inear’</td></tr>
<tr><td>equal variances</td><td>‘equal variances|equal variance|equality of variance’</td></tr>
<tr><td>homogeneity of variances</td><td>‘homogeneity of variances|homogeneity .* variance|
homogene*o*us variance|[^a-z]hov[^a-z]’</td></tr>
<tr><td>sphericity</td><td>‘sphericity’</td></tr>
<tr><td>normal distribution</td><td>‘normal distribution|normal distribut|normality assumption|normally distribut|
assumption of normal|gaussianity’</td></tr>
<tr><td>multivariate normal</td><td>‘multivariate normal’</td></tr>
<tr><td>missing completely at random</td><td>‘missing completely at random|[^a-z]mcar[^a-z]|[^a-z]marc[^a-z]’</td></tr>
<tr><td>missing at random</td><td>‘missing at random|[^a-z]mar assumption’</td></tr>
<tr><td>independency</td><td>‘independency| independence|^independenc[ey]’</td></tr>
<tr><td>orthogonality</td><td>‘orthogonality’</td></tr>
<tr><td>monotonicity</td><td>‘monotonicity’</td></tr>
<tr><td>proportional hazards</td><td>‘proportional hazards|proportional[-]hazard|proportionality assumption| ph assump’</td></tr>
<tr><td>proportional odds</td><td>‘proportional odds’</td></tr>
<tr><td>Weibull assumption</td><td>‘weibull|weibul’</td></tr>
<tr><td>uni dimensionality</td><td>‘uni dimensionality|unidimensionality|uni[-]dimensionality’</td></tr>
<tr><td>local independency</td><td>‘local independency|local independence’</td></tr>
</table>
